# Exploring the thesis experience of Master of Health professions education graduates: a qualitative study

**DOI:** 10.5116/ijme.5abe.2209

**Published:** 2018-04-27

**Authors:** Leslie Skeith, Heather Ridinger, Sushant Srinivasan, Babak Givi, Nazih Youssef, Ilene Harris

**Affiliations:** 1 Division of Hematology and Hematological Malignancies, Department of Medicine, University of Calgary, Canada; 2 Department of Medicine, Vanderbilt University Medical Center, USA; 3 Division of Pediatric Critical Care Medicine, Department of Pediatrics, University of Wisconsin School of Medicine and Public Health, Madison, USA; 4 Department of Otolaryngology, Head and Neck Surgery, New York University School of Medicine, USA; 5 Department of Surgery-Urology, Lebanese American University, Lebanon; 6 Department of Medical Education, University of Illinois College of Medicine, USA

**Keywords:** Health professions, education, theses, qualitative research, USA

## Abstract

**Objectives:**

To explore the thesis experience of recent Master of Health Professions Education (MHPE) graduates in the University of Illinois at Chicago (UIC) program.

**Methods:**

This is a qualitative case study exploring the experience of MHPE graduates between 2014 and 2016 (n=31). Using convenience sampling, all graduates with an email address (n=30) were invited to participate in an online survey and semi-structured interviews. Interviews were completed in-person or via telephone or video conference; interviewers collected detailed notes and audio recordings.  Two authors independently analyzed the data iteratively using thematic analysis and discrepancies were discussed and resolved.

**Results:**

Survey results (n=20, 67%) revealed an average graduation of 5.1 years; 10 graduates (33%) were interviewed. Three themes related to the thesis experience were identified: success factors, challenges, and outcomes. Success factors, when present, promoted completion of a thesis; these included: a supportive program environment, time management, available resources, MHPE foundational coursework, aligning theses with career goals, and identifying a project with limited scope. Challenges made thesis completion more difficult for graduates; these included: institutional factors, personal or professional responsibilities, burnout, externally-imposed deadlines, and barriers in the research process. Despite these challenges, completing the thesis resulted in many professional or personal benefits (outcomes).

**Conclusions:**

Multiple success factors and challenges were identified in the master’s thesis process among MHPE graduates at UIC. These findings can help students conducting education-based scholarship through the master’s thesis process. This study also informs program evaluation and improvements and outlines personal and professional outcomes of completing a master’s thesis.

## Introduction

There is a growing expectation and need for health care professionals interested in medical education to obtain advanced qualifications and pursue education-based research scholarship.^1^^-^^4^ While there are known barriers to training clinician scientists,^5^ little is known about the challenges that Master of Health Professions Education (MHPE) students face when planning and conducting education-based research. By better understanding the MHPE students’ experience when completing education-based research, programs can target interventions to better support the development of future medical educators and scholars.

Training for an academic career in medicine can be challenging. Barriers to clinical research training include how best to integrate clinical training and research, lack of protected time, insufficient infrastructural support for trainees in the research environment, and lack of mentorship.^5^^-^^9^ Practicing medical educators involved in scholarship describe additional challenges such as balancing multiple educator roles, having less well-defined career paths, limited funding for education research, and the emphasis placed on research productivity in academia over other educator roles such as teaching.^1^^,^^2^^,^^4^^,^^10^

Little is known about the barriers that health care professionals face in education-based scholarship as they complete advanced degrees. Students enrolled in advanced MHPE training programs are unique, and often come from a variety of clinical backgrounds with a varying amount of experience in medical education and research training. Learning to conduct education-based research and balancing the ‘day job’ of being a current educator poses unique challenges.

A thesis or research component of an MHPE program is often one of the first exposures that MHPE students have to conducting rigorous education-based research and thus presents an opportunity to observe the successes and challenges that may arise. A master’s thesis project can provide students with the opportunity to synthesize, extend, and apply the knowledge and competencies they have developed during the program, with faculty guidance and mentorship.^3^

The purpose of this qualitative case study was to explore the master’s thesis experience of recent graduates of the University of Illinois at Chicago (UIC) MHPE program. The UIC MHPE Program is one of the most established master’s and doctoral programs in health professions education. The UIC MHPE program offers both online and in-person core and elective MHPE courses and includes a master’s thesis research project that is required to complete the program. The master’s thesis is designed for experiential learning opportunities with education-based research and scholarship. Unfortunately, not all students who are accepted into the MHPE program can successfully complete the thesis and thus time to graduation is significantly prolonged or progress halted.  Not all MHPE programs require a master’s thesis and therefore, a deeper understanding of benefits and barriers to master’s thesis completion among working health professions education students within the UIC MHPE program will not only benefit the UIC MHPE program. However, they can evaluate the characteristics and skills that result in successful completion of a master’s thesis among working health professions education students. Therefore, the primary purpose of this qualitative case study is to explore the thesis experience of recent Master of Health Professions Education (MHPE) graduates in the University of Illinois at Chicago (UIC) program. The results of this study can inform similar programs about the benefits and challenges of incorporating a master’s thesis graduation requirement to support, train and graduate future medical educators and scholars.

## Methods

### Study design and participants

An intrinsic case study inquiry design was chosen to evaluate the unique situation among graduates of the MHPE program. This study format using semi-structured interviews focuses on understanding details of a case to allow for an in-depth exploration of complex issues.^5^ A convenience sample of all UIC MHPE graduates who defended their thesis project between 2014 and 2016 was included (n=31). All graduates with an available email address (n=30) were emailed with a request to participate in an online survey and semi-structured interviews. The UIC MHPE thesis process involves developing a pre-proposal outlining a proposed research project, selecting a thesis committee (2-4 UIC and/or local faculty members to advise on the research process), refining the thesis proposal with the thesis committee, conducting the research, and presenting the thesis in an oral defense followed by written submission in the form of a journal manuscript. The online survey collected demographic information including MHPE enrollment and completion dates, thesis committee members, and publication information. Thesis publication information was supplemented by a literature review.

Graduates who agreed to participate in the online survey (n=20, 67%) were informed that all data would be collected and stored anonymously, and that data would be used in aggregate form by graduating cohort. Graduates who agreed to a semi-structured interview (n=10, 33%) were notified of the study objectives and practices and agreed to audio recording prior to participation. The study was deemed to be minimal risk since participants were former students and the study was being conducted by current MHPE students rather than faculty who could have had undue influence or whose involvement may have elicited social desirability bias. The study was approved by the University of Illinois Chicago Institutional Review Board (IRB).

### Data collection method

The 7-item online survey was designed to collect demographic information about graduates, including the time to graduation, whether the thesis project was published, the number and composition of thesis committee members. It was an anonymous online survey open to any graduate whether or not they agreed to an interview. The semi-structured interviews were conducted in person or via telephone or video conference and were on average 30-60 minutes in length. All interviews were audio recorded. Interview questions (23 items) were developed based on a methodological conceptual framework of the components of thesis completion: 1) selecting a mentor, 2) developing a research question and scope, 3) selecting a research design, 4) navigating the ethics approval process, 5) collecting and analyzing data, 6) writing the manuscript, and 7) defending the thesis. The five interviewers were enrolled MHPE students at the time of the study. As such, interview questions reflected the investigators’ collective inquiries regarding the thesis process. Investigators took detailed notes on a standard interview form during the interview, supplemented the notes, and checked for accuracy by listening to the audio-recordings immediately following the interview to ensure that all ideas were recorded, at which time direct quotations were transcribed verbatim.

### Data analysis

Notes from the interviews were independently analyzed by two investigators (LS, HR) with qualitative analysis experience using a thematic analysis methodology based on an iterative inductive analysis design.^11^ The dataset was comprised of interview notes and audio recordings from each interview. Two investigators independently read the interview notes to generate a list of initial themes. These themes were discussed, and a coding scheme and common definitions were developed. The investigators then re-read and coded the interview records independently, returning to discuss any discrepancies, add subthemes and revise the coding scheme based on a process of constant comparative analysis.^12^ Comparative analysis was performed, and discrepancies were resolved through discussion and reference to relevant quotations and audio-recordings when necessary. All data were collected and stored online in a shared folder to allow all investigators or auditors to examine for dependability. This process continued until thematic saturation was obtained and the dataset had been thoroughly discussed and coded. Themes and subthemes were reviewed and discussed with the remaining investigators, which served as a means of member checking to ensure that the final themes were representative of the interviews that each investigator had conducted.

## Results

Results from the online demographic survey (n= 20, 67% response rate) are presented in Table 1. The mean time from MHPE enrollment to graduation was 5.1 years (range of 2-10). Students’ thesis committees were comprised of a mean of 3.25 members (range of 3-5), and 77% of students' committees included a supervisor from the students’ home institution. Over a quarter of recent graduates had already published their thesis work in a medical education journal.

Three major themes were identified from the thematic analysis, each with multiple subthemes: (1) Success Factors – factors that, when present, facilitated the thesis process and when absent seemed to hinder the thesis process; (2) Challenges - difficulties with the thesis process; and (3) Outcomes - professional or personal benefits gained from thesis completion. Each theme was mentioned by all the interviewed graduates although the subthemes varied in prevalence.

### Success factors

All interviewed graduates commented on one or more of the success factors that helped them succeed in completing their master’s thesis and results with representative quotes are presented in Table 2. Several success factors were identified that supported student progress toward successful thesis completion when present and, when absent, hindered student progress. Subthemes include a supportive program environment; time management strategies; use of available resources; MHPE coursework as a foundation; aligning their thesis topic with career goals and developing a specific research question with limited scope.

**Table 1:** Demographic information of recent MHPE graduates

Demographic			
Year of Graduation^*^		n	%
	2014	8	26
	2015	14	45
	2016	9	29
Years to MHPE completion^‡^		5.1 (2-10)	
Mean committee members^‡^		3.25 (3-5)	
Committees with a local supervisor^*^		24	77
Thesis published in a journal^*^		8	26
	Health Professions Education journal	7	87.5
	Medical specialty journal	1	12.5

^*^Data was based on program information or a literature search for published manuscripts (n=31)

^‡^Data based on survey respondent information (n=20)

### Supportive program environment

Mentorship was the most critical success factor described by all graduates in providing guidance and enriching the experience. Two (10%) graduates described a failed mentorship experience before they were able to succeed with another mentor(s). When selecting thesis committee members, the majority of graduates (90%) considered personal qualities in addition to content expertise. Half of the graduates communicated with a central advisor before deciding on a thesis topic and committee members; one member of the MHPE faculty who was endearingly described as the “faculty mom” provided support and guidance to MHPE students throughout the thesis process. Graduates described feeling a sense of family and collegiality with the mentors, peers and administrative staff who had helped them succeed. The thesis defense was viewed as a "celebration" of their accomplishments, rather than a cause for concern.

### Time management

Seven graduates (70%) mentioned time management as an important aspect of their success. Six graduates (60%) described external accountability, either to committee members or through peer support, as a success factor. Graduates commented that deadlines imposed during coursework were helpful; however once it came to the thesis, “the lack of external deadlines was a huge problem” (No.6, male, 2016 graduate). Students described wanting some form of accountability. As one student commented, “I wish I had set up either a mentor or committee to have more accountability. I think they needed to be more “in your face” and set aside time in my calendar for scholarship” (No.6, male, 2016 graduate).  Students also described techniques for managing their own time. One student used a series of Gantt charts to manage time gradually throughout the year and another set aside vacation time to write the manuscript. Other students sought accountability to each other by having a “thesis buddy” who helped them stay on track.

**Table 2:** Success factors of recent MHPE graduates

Success factors	Subthemes and definitions	Incidence	Representative quotations
Supportive environment	Mentorship: Influence of faculty mentors on the project scope, question, understanding the process, both at UIC or locally	10/10 (100%)	Present: *“The interaction with the mentoring committee and having somebody give that level of interest into what you’re doing. Interest, attention, time, that’s such a gift. I was really grateful to have an excuse to ask for that. In a way, because I’m requiredto. Any other time I’ve desired that sort of thing it sort of feels like an imposition or I’m too nervous to ask. It kind of gives you an excuse to work with experts that I don’t otherwise get easily. It helped overcome some of those barriers that I’ve had to that sort of mentorship outside of a formal program.” *(No.9, female, 2015 graduate) Absent:* “I had a struggle with my first supervisor. He described his strategy as “benign neglect” in a comical way. He wouldn’t reach out to you if you wouldn’t reach out to him and I knew myself that I needed someone who is gonna crack the whip every once in a while, and keep me on track.”* (No.2, female, 2014 graduate)
	Central Advisor: Assistance or thesis advice provided by the program director	5/10 (50%)	*“[Senior faculty member] was the faculty “mom” and she would tell me occasionally that it was time to start thinking about my thesis.”* (No.7, female, 2014 graduate)
	Expectations: Clear expectations about the process and timing of thesis deadlines, defense (including the thesis handbook)	7/10 (70%)	Present: “*Every 6 months you have to submit an update of where you are in the thesis once you start. And it is very simple and does not take long to do it but looking at the mile markers about what they expect was very helpful.” *(No.6, male, 2016 graduate) “*Process is pretty simple and clear – there is a thesis manual, so if you skim it and then read it in more detail when you need to, then it is no problem at all.”* (No.7, female, 2014 graudate) Absent:* “I didn’t understand what a thesis was, especially as a PT (not MD).”* (No.5, female, 2015 graduate) *“It was my first thesis; so many things were unclear of course. So, I had to revise. There is one manual for thesis development by UIC and also I had to review many other theses to see how to write them.”* (No.1, female, 2015 graduate)
	Students: Peer support provided from student to student, networking among peers	5/10 (50%)	*“Rely on classmates, because they will help you make it through.”* (No.4, female, 2016 graduate)
	Thesis Defense: Preparation for defense and/or sense of collegiality	9/10 (90%)	*“[I was] in front of a group of incredibly supportive people who have a vested interest in seeing [me] succeed. It was great to have everybody in the room and show off this thing that you worked so hard to put together.”* (No.4, female, 2016 graduate) *“[The defense] was warm and welcoming and had I known then how it would be I would have looked forward to it more.”* (No. 7, female, 2014 graduate) *“It was really like a celebration. I just felt like a presentation of my project and answering questions. Someone said you are going to know more about it than anyone else in the room, and that was true. I didn’t need to be nearly as nervous as I was.”* (No.9, female, 2015 graduate)
Time Management	Accountability: Accountability and deadlines imposed externally, with mentors or others	3/10 (30%)	Present:* “My new supervisor would meet with me and set deadlines and how things got done. Making it a priority, setting clear goals and being accountable to your supervisor to meet those goals.”* (No.2, female, 2014 graduate) Absent:* “One thing that I missed about the coursework… I had no trouble with the coursework because of the deadlines... The lack of external deadlines was a huge problem... I needed to come up with deadlines and it was very hard to do that.”* (No.6, male, 2016 graduate)
	Setting Aside Time: Setting aside time for thesis completion, either in blocks or gradually	7/10 (70%)	*“At the end of the day, you just need to tell yourself you’re going to get it done.”* (No.10, female, 2016 graduate) *“I would have wanted a more formal plan for communication and how to get the project done. Ultimately did a Gannt chart for process for organization. Even if I got off track, was able to jump back in and get on track and keep track of deadlines.”* (No.5, female, 2015 graduate) *“I took vacation time - that was ABSOLUTELY important… I actually went away to a hotel … it was helpful to get away from the family and from the office at work because of all the interruptions.” *(No.6, male, 2016 graduate)
Available Resources	Help: Hired or volunteer help in the process of thesis completion	9/10 (90%)	*“The only cost that I had -- my daughter videotaped it, bribed with concert tickets, bought bagels for actors.”* (No.4, female, 2016 graduate)
	Electronic: Electronic resources used during research or writing process	8/10 (80%)	*“Sometimes a big kitchen table and a bunch of sticky notes can be just as effective as [coding software]. We used google docs to share the code back and forth.”* (No.10, female, 2016 graduate) *“My main friend was Google.”* (No.1, female, 2015 graduate)
	Prototype Use: Seeking out examples of completed theses or attending a defense	5/10 (50%)	*“I went to articles that I had found as well-written in the past and also looked at colleagues’ articles to see how they get organized ...to have a kind of concrete example.”* (No.3, male, 2016 graduate) *“I recommend going to a thesis defense early as soon as you can to give an idea of scope.”* (No.5, female, 2015 graduate)
	Grant Funding: Funding secured for costs incurred during thesis research	4/10 (40%)	*“I got a small grant from my hospital for transcription of the interviews and paid for the tape recorder.” *(No.2, female, 2014 graduate)
	Other: Other listed resources	3/10 (30%)	*“I actually bought two dissertation handbooks.”* (No.5, female, 2015 graduate)
Coursework as a Foundation	Courses: Individual courses or coursework, in general, influenced the thesis	9/10 (90%)	*“In a lot of the coursework that I did, I used that coursework to build my thesis. The qualitative methods course was also very helpful, because some of the data was qualitative. But all of the courses were very important.” *(No.4, female, 2016 graduate) *“In fact all the courses were instrumental in this thesis because I could use the knowledge acquired in each course.”* (No.1, male, 2015 graduate)
Career Alignment with Thesis	Experience: Job responsibilities align with the thesis process. The job requires a completed thesis/degree.	2/10 (20%)	*“The question came out of my experience as a teacher. I could not find good materials for phase 1 of TBL and we could not find good quality preparation materials...So I was going to develop some videos and I wanted to make it ‘count twice.’”* (No.6, male, 2016 graduate)
	Program of Research: Students are interested in using the thesis to meet their long-term research goals in a specific subject, becoming part of the research network in subject area	4/10 (40%)	*“Your thesis should be something that you’re interested in. It’s your chance to really play and think through different techniques and learn different things. It’s easy to just get it done, but it’s not necessarily something you’re really proud of if you don’t try to think through what it is that makes you interested. I think finding something that makes you think, keeps you passionate, keeps you excited to find the answer will get you closer to finishing. If you’re bored about the topic by the time you finish your proposal, it’s probably the wrong project.” *(No.10, female, 2016 graduate)
	Educational Goals: Thesis process meets personal or educational learning goals	5/10 (50%)	*“I thought, well maybe I should do something that, what I perceived, was going to be harder but would have the benefit of learning from the process...And I’m glad that I did that. It expanded my horizon.” *(No.6, male, 2016 graduate)
Research Question Development	Experience Based: Research question derived from practical to day-to-day work	6/10 (60%)	*“Research question was based on day to day work I was already doing. I kept a running list of things I was curious about. I was doing a lot of work on CSR …[I] might as well make it my project.” *(No.4, female, 2016 graduate)
	Educational Goals Based: Research question was based on wanting to learn new methodologies or techniques in health professions education	3/10 (30%)	*“I wanted to learn different techniques. Someone at my local site who’s a Med Ed researcher said that the advantage of your masters is to go and be interested and learn different techniques and add them to your toolbox. I took that to heart. I balanced between both qualitative and quantitative approaches -- both measurement and exploratory techniques.” *(No.10, female, 2016 graduate)
	Feasibility of Project: Research question related to being able to achieve desired outcomes	2/10 (20%)	*“Picking a project where I had control. I didn’t have to navigate a lot of politics to get this implemented. I didn’t want to get hung up about things out of my control - I saw that happening to other people.” *(No.9, female, 2015 graduate)
Research Question Scope	Scope Stayed Small: Student intentionally tried to keep the scope small	3/10 (30%)	*“Pick a smaller and achievable project. The types of smaller projects that are presented at the MHPE conference are just right. Don’t bite off too much. Picking a project of a small or moderate scale is KEY!”* (No.7, female, 2014 graduate) *“I knew that I have to pick a small, doable project: ‘small is beautiful.”* (No.2, female, 2014 graduate)
	Scope From Big to Small: Starting too big and narrowing later	5/10 (50%)	*“You can come up with some very grand ideas, but then when you start digging down, the devil is in the details. When you start digging down, you realize, wow, just answering specific aim one is five specific aims within it. Because your understanding of the methodology is limited. It’s not until you start diving into the methods you’re going to use to answer this question that you start realizing, holy cow, this is a life’s worth. This is not just a single question.” *(No.8, male, 2016 graduate)
	Enlarging Scope: Scope kept getting larger over time	4/10 (40%)	*“The scope was ginormous. I think because I am relatively experienced with scholarship already, having done residency research projects and numerous other projects on the side, I don’t think my committee felt that they needed to hold me back and stop me from overreaching. So, in fact they probably egged me on and added parts.” *(No.10, female, 2016 graduate)
	Mentorship Help with Scope: Mentorship assistance in narrowing or broadening the scope of the project	7/10 (70%)	*“Wanted to [study] medical students and residents, but the committee said ‘No! You are going to focus on students only.’ They told me that it was too much and I’m glad they did.” *(No.6, male, 2016 graduate)

### Use of available resources

The resources that students used to aid in their thesis completion tended to be simple, widely-available resources such as Microsoft Excel and Word, and Google. One student commented about keeping data analysis simple:

“Sometimes a big kitchen table and a bunch of sticky notes can be just as effective [as coding software]”. (No.10, female, 2016 graduate).

Interestingly, 40% of graduates secured a grant, often a small sum of money from their home institution, to buy supplies or recruit participants. Obtaining a grant is not required. However most (90%) graduates used some hired or volunteer help. Half of the graduates mentioned seeking out a prototype to understand the process and aid in their thesis completion, whether attending a thesis defense or using a competed thesis as a “worked example.”

### The importance of MHPE coursework as a foundation

The majority (90%) of graduates felt that their coursework

positively contributed to thesis preparation. One student commented:

“One of the biggest things that I learned from MHPE was the idea of the conceptual frameworks and how to not just describe what you did but also how to elevate it and to contribute it to the literature”. (No.2, female, 2014 graduate)

The importance of idea generation during the MHPE coursework and using “every course to build your thesis” (No.4, female, 2016 graduate) was described.

### Aligning thesis topic and research question with career goals

Half of the graduates (5, 50%) perceived that the thesis experience would help them to achieve their educational goals and were motivated to make the thesis meaningful in their personal or professional development. A smaller percentage of graduates (4, 40%) used the thesis experience to jumpstart research careers by becoming a content expert; fewer (2, 20%) felt that the thesis was practical (or required) for their job responsibilities. Interestingly, when deciding on a research question, the majority of graduates (6, 60%) chose research topics based on the practicality and applicability to their work, to make the thesis “count twice.”  Others chose their research question based on project feasibility (2, 20%) or as a challenge to learn new methodologies (3, 30%).

### Developing a specific research question with limited scope

Limiting the scope of the thesis research project was an outstanding success factor described by the majority of MHPE graduates. Although they often didn’t realize it until the project was underway, five of the graduates (50%) admitted to starting with a research design scope that was too broad. With the help of mentors, all of these graduates narrowed the scope in some way to successfully complete the thesis. As one student commented,

“I wish I’d have known it could have been smaller. I didn’t have to do everything in one fell swoop. [Georges] Bordage always says, ‘small is beautiful.’ Try to take the first bite of the donut, not shove the whole thing in. I think that would have been liberating. I agonized a lot about coming up with something that was huge and ambitious”. (No.9, female, 2015 graduate)

### Challenges

Several challenges that MHPE graduates experienced that stalled the thesis process included: institutional factors, personal or professional responsibilities, burnout, externally imposed timelines and frustrations with the overall research process.

### Institutional factors

Institutional factors, such as institutional policies at either home institutions or UIC led to frustrations in 70% of graduates. Interestingly, 5 (50%) found the IRB process easy, 3 (30%) found it difficult and 2 (20%) had a mixed experience with the UIC and home institution IRB. One common frustration was the UIC thesis formatting policies, as one student described,

“Crossing your T’s and dotting all your I’s… that was annoying! That felt like busywork. [The thesis manual] has not been updated in years, so it has instructions for using a typewriter. I had PDFs and trying to put them in there was frustrating. That had NEGATIVE educational value”. (No.6, male, 2016 graduate)

Still, other graduates describe lack of local faculty support (3, 30%) or lack of funding (1, 10%) as challenging.

### Professional or personal responsibilities

Work responsibilities were the most common reason (4, 40%), outside of the research itself, that led to taking longer for thesis completion. As one student commented,

“The minute they got word I was interested in [medical education], suddenly I was on every committee and being asked to participate. That’s what drowned me, [and] why I couldn’t get the thesis done. There is a lot of hunger for people with expertise in medical education in medicine right now... so be careful about what you say yes to”. (No.8, male, 2016 graduate)

Other graduates (3, 30%) described personal or family responsibilities as obstacles for completing the thesis in the timeline that they had originally intended. One student describes thesis completion after maternity leave:

“The biggest barrier is once you are back in your normal life it’s not on top of your priority list”. (No.2, female, 2014 graduate)

### Burnout

Two graduates (20%) felt a sense of burnout after completing the coursework, which stalled their thesis progress for lack of motivation. Still others (3, 30%) commented that they had short external deadlines to meet that required them to complete the degree quickly, such as starting fellowship or a new job. While graduates viewed these situations as challenging due to burnout, they typically did not struggle with time management compared to graduates who had no such external deadline.

### Research process

Many (7, 70%) graduates described at least one frustration or challenge with the research process, including recruitment, data collection and analysis and project feasibility. The most frequently expressed challenge within research was data collection and recruitment of participants (4, 40%). Skills learned through coursework mitigated some of these challenges.  One student commented that her data analysis was so difficult that she “could have taken [the] stats course for the time it took [her] to figure out Excel” (No.9, female, 2015 graduate). Thirty percent (3, 30%) of graduates found manuscript writing difficult; some were graduates whose first language was not English. Others found that manuscript writing was the easiest component (6, 60%) of the thesis process, in part due to the preparatory work of the pre-proposal and proposal documents. 

### Outcomes

Graduates reported several important outcomes as result of successfully completing their MHPE thesis. Forty percent of graduates were promoted or took on new responsibilities during the program that they directly attributed to the MHPE program or thesis component. Of the 31 graduates, 26% (8/31) have successfully published their theses in peer-reviewed journals. While none of the ten graduates had published their thesis, the majority (8, 80%) were in the process of revising for submission to a journal or had intentions to publish. As one student commented,

“I will consider it a personal failure if I don’t publish it!” (No.6, male, 2016 graduate)

Other positive aspects of the thesis process included receiving respect and recognition (4, 40%), networking opportunities (5, 50%), self-confidence (7, 70%) and a sense of completion (5, 50%).  The majority of graduates (8, 80%) describe being able to apply their knowledge or skills to new problems and to create scholarship opportunities as a result of their thesis and MHPE experience. As one student commented,

“I think it really forced me to apply what we learned in the coursework. It’s easy when you do the projects in the coursework, and you have to theoretically create a curriculum or theoretically create a research project. It’s really different to actually go out and do it. It was much harder, but I could do it. I really did have the tools to do this successfully from the coursework and with the right support. It was really successful. It’s amazing to me how much I’ve learned and how much I can contribute to the people I’m working with now and how they view what I’ve done as this amazing thing that gives me all sorts of expertise and credibility. I didn’t realize the influence that would have and how important it would be for my career advancement. That’s definitely the most rewarding part”. (No.2, female, 2014 graduate)

## Discussion

Graduates reflecting on their MHPE thesis experience identified several important success factors, challenges, and outcomes to thesis completion. Graduates felt that thesis completion, and thus their MHPE degree, influenced their promotion, led to significant networking opportunities and helped them apply principles learned within the coursework to contribute to the body of literature in health professions education, all of which earned them respect and resulted in improved self-confidence. Several success factors and challenges were also identified to prepare future MHPE graduates and foster MHPE program improvement better.

Strong mentorship, a supportive environment, and external accountability were common factors identified for successful thesis completion. When choosing a research supervisor, matching mentors' relationship styles or personal qualities may be of equal importance to matching content expertise.^13^ More than one mentor is often required to assist early-career investigators.^14^ Having a diverse faculty base promotes the importance of a mentorship model that can best match faculty to MHPE students’ needs. One challenge in distributive learning (distance) master’s program may be the initial thesis supervisor selection and fostering a mentorship relationship from afar.^15^ A central thesis advisor may mitigate this challenge because he or she is familiar with the faculty and can facilitate pairings from a distance. One success factor for thesis completion was faculty and student camaraderie, with benefits including idea generation, sharing resources and external accountability. Giddings and colleagues report the success and positive experiences of a peer-support working group to aid in thesis completion among nursing and midwifery students.^16^ Developing more formal online peer networks may improve accountability and information sharing for thesis completion.

The majority of graduates had to recalibrate their project scope and expectations with mentor assistance once they realized the scope of their initial research question. Most acknowledged that by limiting the project to one, focused research question they were able to finish the project and learn significantly during the process. Targeting mentorship during the planning stage may improve the thesis experience and outcomes.

Challenges to thesis completion included institutional factors, personal or professional responsibilities, burnout, externally imposed timelines, and frustrations with the overall research process. By using the MHPE thesis project as a case example for completing education-based research, we identified barriers that have previously been reported by practicing medical educators. Lack of institutional support for education-based research, balancing different roles and responsibilities of an educator, and lack of funding were identified challenges that are not unique to MHPE students.^1^^,^^2^ Navigating the research process itself was daunting for some students, which has been reported among junior doctors interested in pursuing medical education, as well as among practicing medical educators.^2^^,^^17^ While many challenges may be difficult to modify; others are potential targets for programmatic intervention. Most participants completed their project using widely-available familiar software; however, a majority required some help and/or funding to cover incurred costs. Programs could consider promoting and advertising small funding opportunities to help assist students with thesis-related expenses.

A critical aspect of the graduate thesis experience is the complex social-cultural interactions, which promote identity formation as a medical educator.^17^^,^^18^ Completing an MHPE thesis and program is also an introduction and “transformation that comes through immersion in the medical education environment and association with mentors, teachers, and students with similar interests can be life-changing.”^3^ MHPE graduates did talk about the importance of mentorship, a supportive program environment with the thesis viewed as a celebration, and the importance of building a future network of educators, which does relate to a larger community of practice that may affect their identity formation as educators.^19^

### Limitations

Our sample was restricted to graduates from a single MHPE program, limiting generalizability to other programs. Direct comparison to graduates from a program without a thesis component was not possible. Interviews were limited to a convenience sampling and were not selected randomly, creating the potential for selection bias. Interviewing MHPE students who have not completed the thesis could provide valuable insight into additional barriers or challenges. Potential researcher bias is also another limitation since the authors were (at the time of the study) enrolled MHPE students in various stages of thesis completion. Including data from faculty and administrators may enhance knowledge about the experience and outcomes of thesis completion. Interviewing recent graduates provides the most relevant and timely feedback; however, their experience with publishing thesis projects is limited. Future studies could elicit objective data regarding graduates’ promotion status and a number of peer-reviewed publications compared to graduates from similar programs without a thesis component.

In summary, we present a qualitative study of the thesis experience of recent MHPE graduates from an established master’s program. We found that although completing a thesis project was challenging; graduates found it instrumental in their career development as medical educators. We identified both success factors and challenges to the thesis process that may assist current and future MHPE students. Some of the challenges faced have been previously described by practicing medical educators and may play a role in future identify formation as medical educators. Medical educators or health professions education programs should consider the benefits to career development and scholarship as well as the pitfalls of requiring a thesis component within health professions education graduate programs. Programs that choose to implement a thesis requirement would be wise to include a structured program of accountability and mentorship that facilitates completion and promotes professional development.

### Acknowledgments

We would like to thank the UIC MHPE graduates for participating in our study.

### Conflict of Interest

The authors declare that they have no conflict of interest.
